# Surgery

**Published:** 1859-01

**Authors:** 


### SURGERY.

### II.—*On Polypus of the Œsophagus.* By PROF. A. H. MIDDELDORPFF, of Breslau.

The interesting monograph of Prof. Middeldorpff on polypus of the œsophagus, contains a complete resumé of the state of the science on this subject. We extract from it the following highly interesting case, observed by the author:—

Jos. Jaensch, born in 1811, lost accidentally, when young, the left eye, and a few years later, the left arm. He afterwards enjoyed good health, abstaining from excess of food and drink, and avoiding, particularly, irritating substances. In the spring of 1851 he contracted a violent catarrh, which rendered him deaf for a long time. A catarrhal inflammation of the pharynx remained, and embarrassed deglutition, particularly that of dry bread; excretion of pituitous liquid between the meals; sensations of pressure at the epigastrium and behind the sternum; painless dysphagia, but gradually increasing, very marked in certain positions of the patient, and accompanied by eructation, cough, and dyspnœa; the difficulty of deglutition daily augmented; finally, the ingestion of liquid aliments was alone possible. During an attack of violent cough, the patient once vomited sanguinolent mucus.

At the end of the year 1852, the trouble of deglutition having reached its intensity, he drank water copiously, and was seized with violent vomiting, during which a body resembling a kidney was raised into the mouth between the teeth. An intense dyspnœa followed. The patient succeeded in swallowing the body in question, and applied for medical aid. When Prof. Middeldorpff first saw the patient he was pale and much emaciated; the finger, introduced into the pharynx to the posterior wall of the larynx, did not come in contact with any foreign body; a probe, of half a finger's diameter, was passed down in the median line, and in directing it to the right side, an obstacle was found, which could be easily overcome, and was not very deeply seated.

On the 14th of January, 1853, Prof. Middeldorpff directed the patient to take a large quantity of warm water and an emetic. During the violent vomiting which ensued, a turgid and violaceous body appeared between the dental arches—this was the polypus. Prof. Middeldorpff seized it with the forceps of Museux, drew it toward the left commissure of the mouth, in consequence of which the difficulty of respiration was somewhat diminished; a ligature of waxed silk-thread was applied over the tumor, at a level with the base of the tongue. The operation was attended with repeated vomitings and great dyspnœa. The polypus was afterwards divided with the scalpel three-quarters of an inch above the ligature; it became turgescent, of purple color, and a great quantity of blood escaped from it; in a short time its color sensibly diminished. The patient afterwards swallowed the pedicle of the tumor and the ligature, the extremities of which were attached around the left ear; the vomiting, oppression, and dyspnœa ceased immediately; the patient felt very comfortable; it was ascertained, on repeated trial, that slight traction at the end of the ligature produced pain. The patient was ordered not to touch the thread, to use fluid and cold food, and to present himself every second day. Nothing remarkable occurred up to the eighteenth day after the extirpation. At this time the loop of the ligature rose into the mouth; it measured twelve millimètres in diameter, and thus encircled a pedicle of about thirty-seven millimètres in circumference. From this moment the troubles of the patient ceased; deglutition and respiration were unembarrassed; the appetite soon revived, and the patient gained in strength and flesh.

Prof. Middeldorpff saw his patient five years after the operation; his health was then excellent; the excretion of pituitous liquid had ceased. On external examination of the neck nothing abnormal was discoverable; a probe of eight lines diameter still met with a slight obstacle at a level with the larynx; in short, the health of the patient was perfectly established.

*Examination of the tumor.*—The excised tumor measured eight centimètres in length and four in thickness, and weighed about forty grammes; it was cylindrical; its smooth and shining surface presented some inequalities and excoriations which bled easily; it was covered externally by a layer of stratified pavement epithelium; underneath this a layer of conical papillæ was detected; the papillæ were visible to the naked eye, and arranged with great regularity in a spiral around the longitudinal axis of the tumor, and received vessels at their base; underneath the papillary layer the proper substance of the tumor was situated; Prof. Reichert found it, on examination, very vascular, but without nerves; it was composed of connective tissue, in which the cellular elements were still very visible: in other words, a connective tissue not yet arrived at maturity; here and there fat-globules, either free or in cells, were detected; it was, in short, a vascular fibrous tumor, covered with papillæ. By comparing the length of the extirpated portion with that of the remaining portion of the polypus, and by making repeated measurements and explorations in the dead body, Prof. Middeldorpff arrived at the conclusion, that the extremity of the polypus was situated about two inches from the carotid, and that the pedicle was attached at about a level with the larynx. Although these data are merely approximative, they harmonize, in a remarkable manner, with the results furnished by the catheterism.

Prof. Middeldorpff adds, that Dr. John was the first to describe polypi of the naso-pharyngeal papillæ, in his dissertation, *De Polypis Narium;* Varsoviae, 1855. (*De Polypis Œsophagi*, Breslau, and *Gazette Hebdomad.*, Oct. 8, 1858.)

III.—*On Compound Luxations.* By DR. ALBERT SCHINZINGER, of Freiburg.

Dr. Schinzinger gives, in his elaborate treatise, a complete resumé of the views and experiences of ancient and modern authors, and adds to it some original observations of his own. Compound luxations are, according to the author, such in which a perforation of the soft parts has taken place, and they may be complicated by a laceration of larger arteries and nerves. In the first part of the treatise the author gives a detailed report of four cases treated by himself and Dr. Hecker. The following is a short summary of his observations:—

1. *Compound Luxation of the Radio-ulnar Articulation.*—A man fell from a height of thirty-five feet upon the extended palm of his right hand, fracturing the lower end of the radius, half an inch above the joint, and lacerating the radio-ulnar articulation. The articular end protruded through the skin, and a piece an inch in length was removed with the phalangeal saw and Liston's bone nippers. In the course of four months eleven abscesses had formed in the vicinity of the radius; exfoliation of the sequestrum. In the eighth month the patient was so far restored as that the hand and fingers could be extended and flexed.

2. *Compound Luxation of the Knee-joint Forward.*—A man, thirty-five years of age, fell from a bridge into a shallow river. The condyles of the left femur were luxated into the popliteal space, through the lacerated soft parts. Extemporaneous amputation refused; reaction very violent; resection on the twenty-fourth day; cure.

3. *Compound Luxation of the Tibia Forward.*—The patient, forty years of age, fell from a height of sixty feet, lacerating the ligaments and luxating the tibia forward, and the foot outward; reaction moderate. On the fortieth day resection of the articular end of the tibia to the extent of four inches and a half. Cured in four months.

4. *Compound Luxation of the Leg Forward and Outward.*—Laceration of the soft parts; prolapsus of the extensor digitorum communis brevis; protrusion of the fibular end; ankle-joint widely opened, in a girl twenty-five years of age. Resection of the end of the fibula; reposition; gangrene of the soft parts; cure after five months. Two years afterwards she walked on a shoe with a heel one inch high.

In the second part of his treatise the author speaks of the *frequency* of luxation in different joints. Its *occurrence* depends upon the amount of force operating, and upon the character of the joint. *Simple* luxations are frequent in orbicular joints, rarer in hinge joints, and rarest in amphiarthroses; while in the latter the *compound* luxations are more frequent than in the former.

In regard to the *treatment* of compound luxations three questions remain to be decided upon, viz.: whether to reduce, (Paulus Ægineta,) to resect, (Celsus,) or to amputate, (J. L. Petit.)

Before entering into a discussion on this subject, the author describes the *pathologico-anatomical alterations* in the injured parts, and the *reaction* on these as well as on the whole organism, with great precision and detail. Then he considers the *curative results* of the different methods. He finds, in regard to the former point, a striking resemblance between compound luxations and gunshot wounds.

In compound luxations *the consequences of the mechanical force are not expressed merely by the changes of the articular ends, but the amount of injury consists mostly in the propagation of the mechanical influence far beyond the region of the joint.* The articular surfaces of the articulating bones are thrust against each other, slide beyond their facettes and margins, and in consequence of it a contusion is the result; in some places a separation and concussion of the cartilage is produced; this is propagated to the cells of the bony tissue lying immediately underneath, and is followed by infiltration of blood into the same, and the consequent changes. The periosteum is separated from the bone for a distance, the capsule of the joint torn to shreds, the ligaments severed from their insertions, and often a piece of bone broken off. The muscles and tendons in the immediate vicinity are driven asunder by the head of the bone pressed against them; are crushed in some places, and have their fibres torn. Now the skin, stretched to the utmost extent, bursts, the bone protrudes through an irregular, lacerated, gaping wound; comes in contact with air, dust, sand, etc. In consequence of the laceration of the soft parts blood is effused into the cavity of the joint, and infiltrates the muscles and cellular tissue; violent pain and feeling of uselessness of the limb accompany these occurrences. The feeling of strangulation and nausea Larrey endeavors to explain by the injury of the branches of the sympathetic. The joint is numb, and even the whole body stunned; the brain and nervous system received a shock, circumstances which favor the occurrence of gangrene. The local as well as general reaction must necessarily be violent.

After the author has fully considered the circumstances which render the injury so serious, the reaction so violent, and the reduction so difficult, he lays down the *indication for reposition* in the following words: "Reduction is only to be attempted in quite recent cases, if the bone lies yet in the depth of the wound, if the laceration is incomplete, and the bone has remained for the greatest part in its normal connections, or if only a few lines of the articular end are projecting, and this can be brought back without dragging or bruising the soft parts. It is to be remarked, that compound luxations of smaller joints, such as fingers and toes, are frequently followed by tetanus."

In reference to *resection* the author observes that already in ancient times this operation had been proposed or executed, but it was, as Velpeau remarks, practiced chiefly from want of knowledge how to arrest hemorrhage, in order to avoid amputation, but not with the clear comprehension of the principle involved, and of the humane object to save a limb. It was left to the scientific zeal, and humane tendency of some physicians of the second half of last century, to establish more decided indications for the operation, and to introduce it again to the notice of the profession. In England it found its methodic inventors in White, Parke, Crampton, etc., in France its zealous imitators in Moreau and Roux, in Germany in Textor, Jæger, and Fricke.

The most valuable contributions to the already richly endowed chapter on resection, we owe to Prof. Ried, (On Resection of Bones,) and to the industrious statistical researches of Malgaigne.

Making extensive use of the existing literature, the author considers *resection of the particular joints in reference to their statistical results.* The most successful have been resections at the *shoulder-joint*, (Le vrai triomphe de la resection c'est quand on la pratique sur la tête de l'humerus; *Gazette Médicale*, No. 16, 1858.) More difficult, and attended with less success, proved to be resections of the *elbow-joint;* they were, however, at all events, much more favorable than the results of simple and of forced reposition. In the same way the author shows conclusively the superiority of resection at the other joints of the superior extremity over forced reposition and amputation.

He was, however, not so able to defend the use of resection in injuries of the lower extremities, particularly of the *knee-joint*, against the objections of many distinguished surgeons. Sanson, Pétrequin, and before them Guthrie and Dupuytren, and nearly all surgeons of Germany, condemn resection of the *knee-joint*, as it exposes life to greater risk than amputation. Ried, alone, prefers resection to amputation, and has quite recently found approval and imitators among the English surgeons. Jones collected thirty-three resections of the knee-joint, of which five were fatal, and twenty-six terminated in recovery; and since 1830 it was executed in Germany about thirty times, in almost every case on account of *caries* and *necrosis.* Resections after *traumatic injuries* terminated most unfavorably, and they were therefore limited to only a few cases; four of which have been recorded, and proved fatal.

The advantages of resection in complicated luxations of the *ankle-joint* are proved by many cases, and the mode of proceeding was accurately laid down by Cooper. The presence of only one of the articular surfaces will often preserve motion to the limb. Cooper is particularly favorable to the resection of the inferior ends of the tibia and fibula, as he never observed an unfavorable issue of the operation.

In complicated luxations of *the astragalus*, total extirpation of the same is the best measure; statistical reports testify in favor of it by large numbers; Malgaigne quotes eight cases; Turner collected in England forty-six cases, among which were eighteen of total extirpation; of these only thirteen recovered with ability to move the limb. Broca recorded fifty-two cases, of which forty-two ended favorably. Still more encouraging are the known results of resection in compound luxations of the *smaller joints.* The author takes then occasion to speak of the experiments of A. Wagner, on the process of healing after resection of bones, and draws some conclusions particularly in reference to the prognosis of the operation.

The treatment recommended by the author after resection is as simple as rational, and modeled according to the therapeutical axioms of the Vienna school. He speaks with particular praise of the "Railroad Apparatus" of Prof. Dumreicher.

Finally, he considers the question of *amputation.* He calls this measure the *ultima ratio* in compound luxations, and advises it only as a last attempt to save the life of the patient.

The author states the advantages of resection over amputation very clearly in the following points:—

1. The duration of the healing process is much shorter than in reduction.
2. Very dangerous symptoms are avoided, such as muscular contractions, reproduction of the luxation, tetanus, gangrene, articular suppurations of long duration, caries, and necrosis of the articular ends.
3. The proportion of fatal cases is very moderate.
4. Motion is, in the majority of cases, preserved.
5. The operation is less dangerous, and of easy execution.
6. Consecutive amputation is rarely necessary after resection, while it is often required after reduction.
7. Finally, the moral impression of an amputation, compared to that of a resection, is worth consideration, particularly if the amputation of the limb must be performed in the moment, where the patient is overcome by prostration, pain, etc. The idea of preserving a limb, merely shortened a few lines, reassures the patient, consoles him, and makes him bear pains and affliction better.

Dr. Schinzinger's treatise pleads energetically in favor of conservative surgery, and, although it does not bring many new facts or arguments, it is highly interesting as a faithful collection and critical exposé of the experiments made and views entertained, up to the present time, on the subject of compound luxations. (*Die Complicirten Luxationen,* von Dr. A. Schinzinger, Privatdocent an der Universität Freiburg. Lahr: Verlag von Schönberg & Co., 1858; pp. 53.)

